# Sodium Butyrate Alleviates Free Fatty Acid-Induced Steatosis in Primary Chicken Hepatocytes via Regulating the ROS/GPX4/Ferroptosis Pathway

**DOI:** 10.3390/antiox13020140

**Published:** 2024-01-23

**Authors:** Xinyi Cheng, Yang Hu, Xiaoqing Yu, Jinyan Chen, Xiaoquan Guo, Huabin Cao, Guoliang Hu, Yu Zhuang

**Affiliations:** Jiangxi Provincial Key Laboratory for Animal Health, Institute of Animal Population Health, College of Animal Science and Technology, Jiangxi Agricultural University, No. 1101 Zhimin Avenue, Economic and Technological Development District, Nanchang 330045, China; chengxinyi@jxau.edu.cn (X.C.); yanghu2009@jxau.edu.cn (Y.H.); lijingni@stu.jxau.edu.cn (X.Y.); jan2021@stu.jxau.edu.cn (J.C.); xqguo20720@jxau.edu.cn (X.G.); chbin20020804@jxau.edu.cn (H.C.)

**Keywords:** fatty liver hemorrhagic syndrome, sodium butyrate, ferroptosis, steatosis, ferrostatin-1, RSL3

## Abstract

Fatty liver hemorrhagic syndrome (FLHS) in laying hens is a nutritional metabolic disease commonly observed in high-yielding laying hens. Sodium butyrate (NaB) and ferroptosis were reported to contribute to the pathogenesis of fatty liver-related diseases. However, the underlying mechanism of NaB in FLHS and whether it mediates ferroptosis remains unclear. A chicken primary hepatocyte induced by free fatty acids (FFAs, keeping the ratio of sodium oleate and sodium palmitate concentrations at 2:1) was established, which received treatments with NaB, the ferroptosis inducer RAS-selective lethal 3 (RSL3), and the inhibitor ferrostatin-1 (Fer-1). As a result, NaB increased biochemical and lipid metabolism indices, and the antioxidant level, while inhibiting intracellular ROS accumulation and the activation of the ferroptosis signaling pathway, as evidenced by a reduction in intracellular iron concentration, upregulated GPX4 and xCT expression, and inhibited NCOA4 and ACSL4 expression. Furthermore, treatment with Fer-1 reinforced the protective effects of NaB, while RSL3 reversed it by blocking the ROS/GPX4/ferroptosis pathway, leading to the accumulation of lipid droplets and oxidative stress. Collectively, our findings demonstrated that NaB protects hepatocytes by regulating the ROS/GPX4-mediated ferroptosis pathway, providing a new strategy and target for the treatment of FLHS.

## 1. Introduction

Fatty liver hemorrhagic syndrome (FLHS) is a widespread nutritional and metabolic disorder in high-producing egg-laying hens, marked by an inordinate buildup of lipids within their liver tissue [1,2]. This syndrome triggers sudden deaths in the birds and a decline in egg production, thereby causing significant economic detriment to the poultry industry [3]. The prevailing academic consensus maintains that a spectrum of factors, such as dietary imbalances, agricultural management, hormonal disruptions, and toxic exposures, crucially trigger the onset of FLHS [4]. Recent studies have pinpointed high-energy and low-protein (HELP) diets as the fundamental pathological driver behind FLHS [5]. Advanced clinical research has shed light on the intricate interplay of oxidative stress, inflammatory infiltration, and insulin resistance in the genesis of non-alcoholic fatty liver diseases (NAFLD) [6]. Notably, the pathogenesis of fatty liver disease is similar to that of FLHS. Contemporary findings establish a robust link between ferroptosis, a meticulously regulated form of cell death, and the accretion of liver fat, positing the modulation of ferroptosis as a potential treatment avenue for the management of non-alcoholic fatty liver conditions [7].

Ferroptosis is an iron-dependent form of non-apoptotic cell death distinguished by lipid peroxidation, which stands apart from autophagy, apoptosis, and necrosis [8]. Recent studies have revealed metabolic pathways—the guanosine triphosphate cyclohydrolase 1/tetrahydrobiopterin/dihydrofolate reductase axis, the cysteine/glutathione (GSH)/glutathione peroxidase 4 (GPX4) axis, and the ferroptosis suppressor protein 1/coenzyme Q (CoQ) axis—as key modulators in the ferroptosis process [9]. Evidence linking ferroptosis with pathology arose from ischemia/reperfusion injury in hepatic and renal tissues [10]. Subsequent investigations have highlighted the pivotal role of lipid peroxidation-driven ferroptosis across a spectrum of liver diseases, encompassing non-alcoholic fatty liver disease (NAFLD), viral-induced steatohepatitis, and alcoholic fatty liver disease (AFLD) [11,12]. Cui S’s research demonstrated that ferroptosis can provoke inflammatory responses within simple steatosis, precipitating the initiation and progression of non-alcoholic steatohepatitis in vitro (SV589 cell and HT1080 cell) and in vivo (mice) [11]. Moreover, previous studies have indicated that the application of iron chelators and antioxidants could potentially mitigate or reverse the deleterious effects of hepatocellular death, inflammation, and lipid peroxidation associated with NAFLD in mice and humans [13,14]. Targeting ferroptosis emerges as an auspicious therapeutic avenue for an array of liver diseases. Nevertheless, the role of ferroptosis in lipid accumulation within the livers of laying hens requires further investigation to elucidate its impacts and underlying mechanisms.

Butyrate, a principal fermentation byproduct of crude fiber by the animal intestinal microbiome, is a critical modulator of energy metabolism, playing a significant role in reducing hepatic lipid accumulation and alleviating intestinal inflammation [15,16]. Sun B’s studies revealed that sodium butyrate (NaB)-induced peroxisome proliferator-activated receptors α activation stimulates fatty acid β-oxidation and inhibits nuclear factor kappa-B-mediated inflammation pathways via protein–protein interactions, thus contributing to the amelioration of high-fat-diet-induced NAFLD in adult rats [17]. Additionally, a previous study from Zhao ZH’s study suggested that NaB acts as a suppressor of hepatic lipogenesis in NAFLD, attenuating hepatic steatosis, and improving lipid profiles and liver function predominantly via the liver kinase B1-AMP-activated protein kinase (AMPK)-insulin-induced gene signaling pathway in NAFLD mice models [18]. Notably, Zhao LQ’s study found that the dietary supplementation of coated NaB inhibited fat deposition in livers and abdominal fat tissues of broilers, and significantly reduced adipocytic fat accumulation in chicken preadipocytes [19]. Previous experiments have shown that the HELP diet leads to a substantial decline in the levels of short-chain fatty acids in the guts of laying hens, causing FLHS [20]. Moreover, recent studies showed butyrate’s ability to impede the progression of NAFLD by stimulating key metabolic pathways such as AMPK and peroxisome proliferator-activated receptor gamma coactivator-1 alpha. Notably, supplemental with coated NaB efficiently privileges birds’ performance and health [21]. Further investigations suggest that NaB reduces hepatic fat deposition by modulating mitochondrial energy metabolism through SIRT3 in the SD rats model [22].

Consequently, we proposed that butyrate potentially modulates the lipid metabolism pathways within hepatic cells, although its link to the ferroptosis signaling cascade remains to be elucidated, necessitating further investigation. To decode the complexities of FLHS, we engineered an in vitro platform featuring chicken primary hepatocytes subjected to free fatty acids (FFAs). Treatment protocols for these hepatocytes incorporated NaB, the ferroptosis inducer RAS-selective lethal 3 (RSL3), and the ferroptosis inhibitor ferrostatin-1 (Fer-1). Our research revealed that butyrate significantly reduces both lipid deposition and hepatic injury prompted by FFAs in these hepatocytes, deploying its protective arsenal via the reactive oxygen species (ROS)/GPX4/ferroptosis axis.

## 2. Materials and Methods

### 2.1. Chemicals and Reagents

RSL3 (#HY-100218A) and Fer-1 (#HY-100579) were purchased from MedChemExpress (MCE, Monmouth Junction, NJ, USA). Sodium butyrate (NaB, #S1999) was purchased from Selleck Chemicals, Houston, TX, USA. Dimethyl sulfoxide (DMSO, Solarbio, Beijing, China, #D8371) was used to prepare the stock solution of drugs. FFAs was purchased from Shanghai Keshun Science and Technology co., Ltd. (Shanghai, China). Fetal Bovine Serum (FBS, # FSD500) was purchased from Excell Bio. DCFH-DA (#S0033S) and DAPI (#C1002) were purchased from Beyotime Bio (Shanghai, China). Alanine aminotransferase (ALT, #C009-2-1), aspartate aminotransferase (AST, #C010-2-1), total cholesterol (TC, #A111-1-1), triglyceride (TG, #A110-1-1), malondialdehyde (MDA, #A003-4-1), glutathione peroxidase (GSH-pX, #A005-1-2), and glutathione (GSH, #A006-2-1) were purchased from Nanjing Jiancheng Bio (Nanjing, China). Transferrin receptor (TFRC, # bs-41331R), ferritin heavy chain 1 (FTH1, #bs-5907R), nuclear receptor coactivator 4 (NCOA4, #bs-19051R), cyclooxygenase-2 (COX-2, #bs-10411R), GPX4 (#bs-3884R), and GAPDH (#bs-41373R) were purchased from Bioss Bio (Beijing, China). Solute carrier family 7 member 11 (SLC7A11 or xCT, #26864-1-AP) was purchased from Proteintech Bio (Rosemont, IL, USA).

### 2.2. Primary Chicken Hepatocyte Isolation and Culture

The isolation and culture of primary chicken hepatocytes were based on those previously reported by Huang C. et al. [23]. Embryonic chicken livers from 14-day-old embryos were aseptically harvested and maintained in phosphate-buffered saline (PBS) at 4 °C, following which the liver tissue was diced into approximately 1 mm^3^ pieces. Subsequently, the fragments underwent enzymatic digestion using 0.1% collagenase type IV at 37 °C for 15 min. After that, the collagenous was inactivated by an additional DMEM/F12 medium containing 10% FBS. Subsequently, the hepatocytes suspension was filtered with 200-mesh and 400-mesh cell sieves, and the hepatocytes were collected through centrifugation and suspended in DMEM/F12. The isolated primary chicken hepatocytes were then seeded into 6-well or 96-well culture plates at densities of 5 × 10^6^ cells/well in 2 mL or 5 × 10^5^ cells/well in 100 µL DMEM/F12, respectively. Cultures were incubated at 37 °C in a humidified environment composed of 95% air and 5% CO_2_ to facilitate cell growth.

### 2.3. Cell Treatments

FFAs contained 12 mmol/L sodium oleate (OA) and 6 mmol/L sodium palmitate (PA), formulated as 1 mmol/L FFAs (keeping the ratio of OA and PA concentrations at 2:1), to establish hepatic steatosis model in primary hepatocytes [24]. The hepatocytes were subjected to treatment with 1 mM FFAs and/or 0.5 mM NaB for 24 h according to our previous studies. Concurrently, to examine the regulatory effects on ferroptosis, cells were co-treated with the ferroptosis inducer 1 μM RSL3 and the inhibitor 5 μM Fer-1, following dose–response relationships established in previous studies. After a 24 h co-treatment period, hepatocytes were harvested for further experimental analysis. Each experiment was repeated three times.

### 2.4. Oil Red Staining

The post-treatment cells were rinsed thrice with cold PBS, followed by fixation in a 4% paraformaldehyde solution for 30 min. To each well, 500 µL of Oil Red O staining solution was added, and the cells were incubated at 37 °C for 20 min in the dark. After incubation, the oil red O solution was discarded and 500 µL of 60% isopropyl alcohol was added to each well for 15 s before being removed. Subsequently, cells were washed three times with 1× PBS. The stained cells were examined under a microscope and images were captured for documentation. The software Image J (Version. 20) was used to quantitative analyze the lipid droplet (red) in the images, and the lipid droplet identified and counted by randomly selecting three visual fields.

### 2.5. The Determination of TG, TC, AST, and ALT Content

The concentrations of TG, TC, AST, and ALT were quantified using a commercial assay kit provided by Nanjing Jiancheng Biotechnology (Nanjing, China) for the purpose of hepatocyte function. To ensure accurate comparison, TG and TC were normalized to protein concentrations, which were determined using the Bradford protein assay kit from Beyotime Biotechnology, following the manufacturer’s guidelines. AST and ALT were expressed in U/L of culture medium, following the manufacturer’s guidelines.

### 2.6. Transmission Electron Microscopy (TEM) Analysis

TEM observation was performed as described by Cheng X.Y. et al. [25]. Briefly, cells detached using trypsin were subjected to centrifugation at 2500 rpm for 5 min, repeated thrice, followed by fixation in 2.5% glutaraldehyde (prepared in 0.1 M phosphate buffer, Servicebio, Wuhan, China) at ambient temperature for 2 h. Subsequently, cells were rinsed with Sorensen’s phosphate buffer, dehydrated through a graded ethanol series, embedded, and sectioned. Finally, the sections were stained appropriately and examined under the microscope (TEM Zeiss 900, Jena, Germany).

### 2.7. The Determination of Antioxidant Enzyme Activity

The activities of GSH-pX and the contents of MDA and GSH in hepatocytes were quantified using for the purpose of antioxidant function. In brief, the cells with different treatments were collected by PBS and mechanically broken. To ensure accurate comparison, these data were normalized to protein concentrations, which were determined following the manufacturer’s guidelines. Then, the content or viability was calculated by reading on the microplate reader according to the different reaction substrates of the antioxidant kit.

### 2.8. The Determination of Iron Ion Content

The contents of iron in hepatocytes were determined by a commercial assay kit provided by Elabscience Biotechnology (Houston, TX, USA) according to the manufacturer’s guidelines. To ensure accurate comparison, these data were normalized to protein concentrations, which were determined using the Bradford protein assay kit from Beyotime Biotechnology (Shanghai, China), following the manufacturer’s guidelines.

### 2.9. Real-Time Quantitative PCR (RT-qPCR)

Hepatocytes were collected and total RNA was extracted using Trizol reagent. Total RNA (1 μg) was revered into cDNA using HiScript III RT SuperMix for qPCR (+gDNA wiper) (Vazyme, Nanjing, China) according to the manufacturer’s instructions. And then, RT-qPCR was performed as described previously [25]. The relative abundance of gene transcripts was normalized to the values of the control treatment by using the 2^−ΔΔCt^ method [19]. All primers were designed by online designer Primer-BLAST (https://www.ncbi.nlm.nih.gov/tools/primerblast, accessed on 1 January 2023) and synthesized by Tsingke Biotech Co., Ltd. (Changsha, Hunan, China) (Table 1).

### 2.10. Western Blotting

The samples of primary chicken hepatocytes were treated with radio immunoprecipitation assay lysis buffer (Beyotime, Shanghai, China). Proteins quantified using the BCA Protein Assay Kit (Beyotime, Shanghai, China) were separated by SDS-PAGE and transferred onto polyvinylidene difluoride membranes (Millipore, Temecula, CA, USA). The membranes were blocked with 5% BSA in phosphate-buffered solution for 4 h and then incubated with antibodies against TFRC, FTH1, NCOA4, xCT, COX-2, GPX4, or GAPDH overnight at 4 °C. The antibodies were all purchased from Cell Signaling Technology, Danvers, MA, USA. On the next day, membranes were probed with an HRP-conjugated secondary antibody (Beyotime, Shanghai, China). Western blot analysis was performed and quantified with Image J (National Institutes of Health, Bethesda, MD, USA).

### 2.11. Immunofluorescence Staining

Sterilized coverslips were arrayed in 24-well plates, upon which hepatocytes were subjected to a 24 h treatment regimen with NaB, FFAs, Fer-1, and RSL3. Following treatment, the cells were stabilized with a 4% paraformaldehyde solution for 15 min at room temperature, subsequently permeabilized by 0.5% Triton X-100, and then blocked in 5% BSA for 2 h. Subsequently, the hepatocytes were incubated overnight at 4 °C with primary antibodies targeting GPX4 and fluorescent probe DCFH-DA (ROS indicator). Fluorescent secondary antibodies were applied for one hour at 37 °C under light-protective conditions. Nuclear staining with DAPI was performed before glycerin sealing. Fluorescent imaging was executed with a laser scanning confocal microscope (Nikon, Tokyo, Japan). The software Image J was used to quantitatively analyze the fluorescence intensities of DCFH-DA (ROS indicator) in the images, and the fluorescence intensities of DCFH-DA (ROS indicator) were identified and counted by randomly selecting three visual fields.

### 2.12. Statistical Analyses

Statistical analysis between groups was executed using one-way ANOVA via SPSS software, version 25.0. The findings were articulated as the mean accompanied by the standard deviation (Mean ± SD). Graphical representations of the data were meticulously produced using GraphPad Prism, version 9. The benchmarks for statistical significance were set at *p* < 0.05. In the graphical annotations, ‘ns’ signified a non-significant variance with *p* > 0.05, a single asterisk ‘*’ identified significant differences at *p* < 0.05, a double asterisk ‘**’ denoted highly significant differences when *p* < 0.01, and three asterisk ‘***’ denoted highly significant differences when *p* < 0.001.

## 3. Results

### 3.1. NaB Alleviated Fatty Degeneration and Liver Injury in Primary Chicken Hepatocytes Induced by FFAs

**Figure 1:**
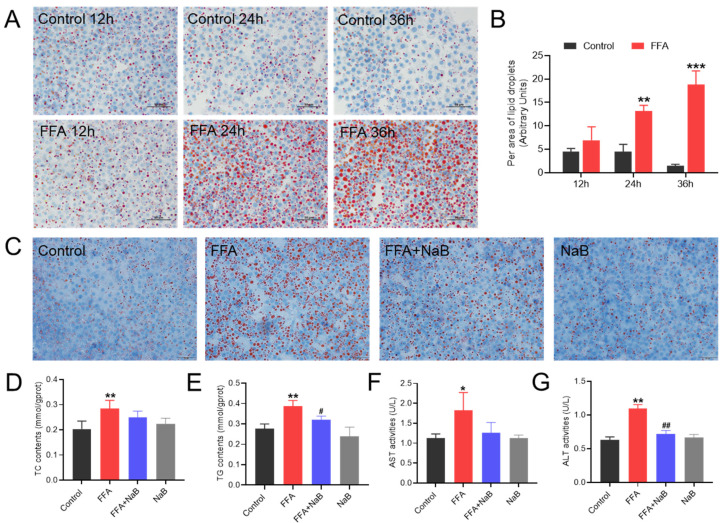
NaB alleviated fatty degeneration and liver injury in primary chicken hepatocytes induced by free fatty acids (FFAs). A total of (**A**) 1 mM FFAs induced lipid droplet accumulation in primary chicken hepatocytes at 12 h, 24 h, and 36 h-post treatment. (**B**) Quantitative analysis of lipid droplets (lipid droplet area ratio was quantified using Image J). (**C**) Effect of 0.5 mM NaB on lipid droplet accumulation in primary hepatocytes induced by FFAs (magnification times, 200×; scale bar, 50 μm). (**D**–**G**) Effect of NaB on TC, TG, AST, and ALT levels in chicken primary hepatocytes induced by FFAs. Data are expressed as the Mean ± SD of at least three independent experiments. An asterisk (*) denotes a statistically significant difference relative to the control group, with * *p* < 0.05, ** *p* < 0.01, and *** *p* < 0.001, indicating different levels of significance, respectively. A hash (#) signifies a significant difference between the specified groups, with # *p* < 0.05 and ## *p* < 0.01 corresponding to different levels of significance, respectively. Each experiment was repeated three times. Below is the same.

To investigate the impact of 0.5 mM NaB on lipid metabolism in primary chicken hepatocytes, we initially isolated these cells from laying hens and induced fatty liver disease using 1 mM FFAs. As expected, 1 mM FFAs could induce time-dependent (12 h, 24 h, and 36 h) lipid droplet accumulation in primary chicken hepatocytes (*p* < 0.01) (Figure 1A,B). In addition, NaB significantly decreased the lipid droplet accumulation in FFAs-induced hepatic steatosis. Furthermore, NaB at the dose of 0.5 mM intervention could significantly decrease the levels of TG and TC in hepatocytes induced by FFAs, thereby lessening the accumulation of lipid droplets in liver cells (Figure 1D,E). It has been discovered that NaB effectively ameliorated the lipid damage in hepatocytes caused by FFAs as evidenced by the significantly decreased activities of ALT and AST in cellular supernatant (Figure 1F,G).

### 3.2. NaB Attenuated Mitochondrial Injury by Reducing ROS Levels and Upregulated Antioxidant Defenses in Hepatocytes Induced by FFAs

**Figure 2:**
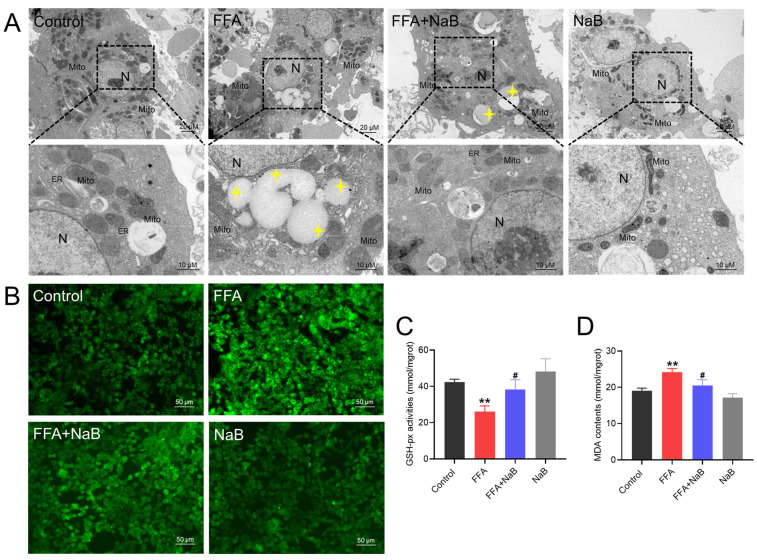
NaB attenuated mitochondrial injury by reducing ROS levels and upregulated antioxidant defenses in hepatocytes induced by FFAs. (**A**) Transmission electron microscopy observation (N: nucleus, Mito: mitochondria, ER: endoplasmic reticulum, yellow four-pointed star: lipid droplet), scale bars: 20 μm and 10 μm. (**B**) Immunofluorescence images of the fluorescent probe DCFH-DA (ROS indicator, green) staining of representative cellular sections from various treatment groups, scale bar: 50 μm. (**C**,**D**) Effect of NaB on GSH-px activity and MDA content in chicken primary hepatocytes induced by FFAs. An asterisk (*) denotes a statistically significant difference relative to the control group, with ** *p* < 0.01, indicating different levels of significance. A hash (#) signifies a significant difference between the specified groups, with # *p* < 0.05 corresponding to different levels of significance.

To investigate the metabolism through which 0.5 mM NaB mitigates lipid accumulation induced by 1 mM FFAs in primary chicken hepatocytes, transmission electron microscopy was employed to evaluate subcellular structures after various treatments. Accumulation of lipid vacuoles in the cytoplasm of hepatocytes, and mitochondria exhibited morphological disruption following the FFAs treatment, wherein mitochondrial sizes were slightly increased, and cristae were disrupted. Treatment with NaB was found to mitigate the formation of lipid droplets and mitochondrial damage in hepatocytes induced by FFAs (Figure 2A). Further analysis of the ROS and antioxidant enzyme indices showed that NaB significantly reduces ROS accumulation in FFAs-induced hepatocytes, enhances GSH-pX activity, and consequently decreases MDA content (*p* < 0.05) (Figure 2B–D).

### 3.3. NaB Upregulates GPX4, Mitigating Iron Accumulation and Disrupting the Signaling Cascade of Ferroptosis in Hepatocytes Induced by FFAs

**Figure 3:**
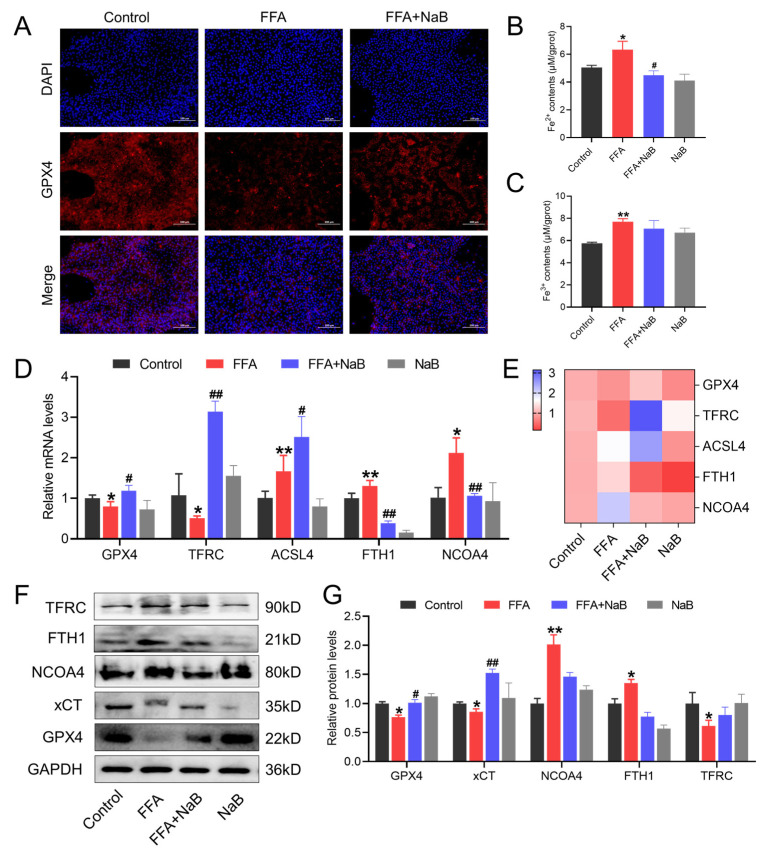
NaB upregulates GPX4, mitigating iron accumulation and disrupting the signaling cascade of ferroptosis in hepatocytes induced by FFAs. (**A**) Immunofluorescence images of the fluorescent GPX4 (red) and DAPI (blue) staining of representative cellular sections from various treatments. (**B**,**C**) Effect of NaB on Fe^2+^ and Fe^3+^ levels in chicken primary hepatocytes induced by FFAs. (**D**,**E**) Effect of NaB on the mRNA levels of GPX4, TFRC, ACSL4, FTH1, and NCOA4 in primary chicken hepatocytes induced by FFAs. (**F**,**G**) Effect of NaB on the protein levels of GPX4, xCT, NCOA4, FTH1, and TFRC in primary chicken hepatocyte induced by FFAs. An asterisk (*) denotes a statistically significant difference relative to the control group, with * *p* < 0.05 and ** *p* < 0.01, indicating different levels of significance. A hash (#) signifies a significant difference between the specified groups, with # *p* < 0.05 and ## *p* < 0.01 corresponding to different levels of significance.

Within the framework of ferroptosis—a form of cell death hallmarked by oxidative stress and lipid peroxidation—the influence of 0.5 mM NaB on 1 mM FFAs-triggered hepatocellular ferroptosis was scrutinized. NaB significantly elevates the expression of GPX4 in hepatocytes induced by FFAs, a protein previously demonstrated to negatively regulate ferroptosis (Figure 3A). Moreover, against the backdrop of ferroptosis being an emergent paradigm of iron-dependent cell demise with ions at its fulcrum. As shown in Figure 3B,C, NaB was observed to significantly diminish the contents of Fe^2+^ and Fe^3+^ in FFAs-stimulated hepatocytes. Further scrutinizing the gene expression profiles pertinent to the ferroptosis pathway (GPX4, TFRC, ACSL4, FTH1, and NCOA4) showed that NaB deters the ferroptosis signaling cascade in hepatocytes induced by FFAs (Figure 3D–G).

### 3.4. The Ferroptosis Agonist RSL3 Abrogated the Ameliorative Impact of NaB on Lipid Accumulation in Hepatocytes Induced by FFAs

**Figure 4:**
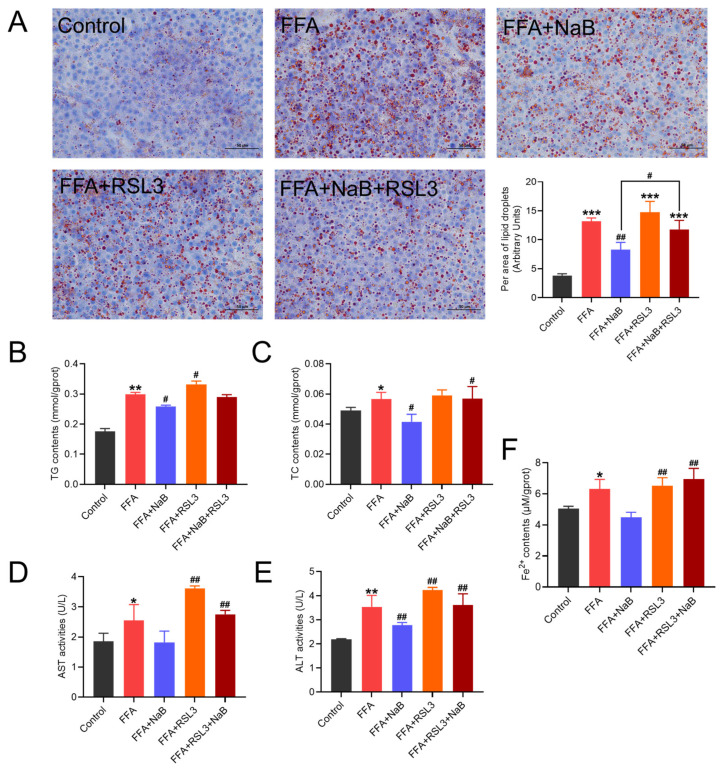
The ferroptosis agonist RSL3 (1 μM) abrogates the ameliorative impact of NaB on lipid accumulation in hepatocytes induced by FFAs. (**A**) Effect of RSL3 and/or NaB on lipid droplet accumulation in primary hepatocytes induced by FFAs (lipid droplet area ratio was quantified using Image J). (**B**–**F**) Effect of RSL3 and/or NaB on the contents of TG and TC, the activities of AST and ALT, and Fe^2+^ content in primary chicken hepatocytes induced by FFAs. An asterisk (*) denotes a statistically significant difference relative to the control group, with * *p* < 0.05, ** *p* < 0.01, and *** *p* < 0.001, indicating different levels of significance. A hash (#) signifies a significant difference between the specified groups, with # *p* < 0.05 and ## *p* < 0.01 corresponding to different levels of significance.

To investigate the role of ferroptosis in 0.5 mM NaB-mediated lipid metabolism in 1 mM FFAs-induced hepatocytes, the ferroptosis agonist 1 μM RSL3 was utilized to assess its impact on NaB intervention in lipid droplet accumulation in FFAs-stimulated hepatic cells. As depicted in Figure 4A, FFAs significantly increased the accumulation of lipid droplets (*p* < 0.01), and RSL3 further effectively enhanced the accumulation of lipid droplets in primary chicken hepatocytes induced by FFAs (*p* < 0.01). Intriguingly, RSL3 completely negated the reversative effect of NaB, as evidenced by the increasing levels of TC and TG (Figure 4B,C). Moreover, RSL3 effectively counteracted the protective effect of NaB on FFAs-induced hepatocellular damage, demonstrated by the significantly increased levels of ALT and AST (Figure 4D,E). In addition, RSL3 could lead to the accumulation of cellular Fe^2+^ (Figure 4F).

### 3.5. RSL3 Abrogated NaB-Induced Antioxidative Response and GPX4 Expression in FFAs-Stimulated Hepatocytes, Exacerbating GPX4-Dependent Ferroptosis

**Figure 5:**
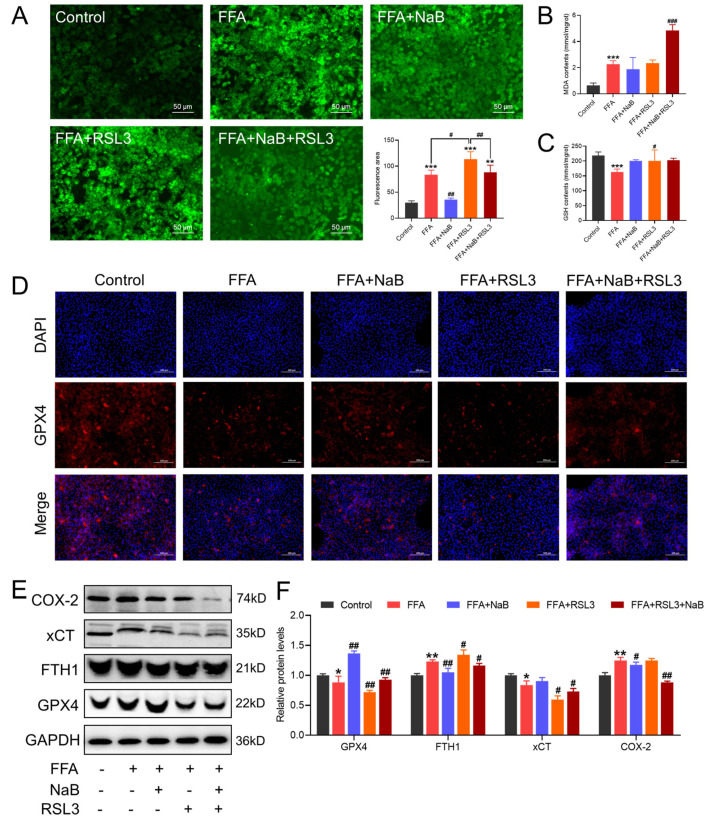
RSL3 abrogates NaB-induced antioxidative response and GPX4 expression in FFAs-stimulated hepatocytes, exacerbating GPX4-dependent ferroptosis. (**A**) Immunofluorescence images of the fluorescent probe DCFH-DA (ROS indicator, green) staining of representative cellular sections from various treatments (green fluorescence intensity was quantified using Image J). (**B**,**C**) Effect of RSL3 and/or NaB on the contents of MDA and GSH in primary chicken hepatocytes induced by FFAs. (**D**) Immunofluorescence images of the fluorescent GPX4 (red) and DAPI (blue) staining of representative cellular sections from various treatments. (**E**,**F**) Effect of RSL3 and/or NaB on the protein levels of COX-2, xCT, FTH1, and GPX4 in primary chicken hepatocytes induced by FFAs. An asterisk (*) denotes a statistically significant difference relative to the control group, with * *p* < 0.05, ** *p* < 0.01, and *** *p* < 0.001, indicating different levels of significance. A hash (#) signifies a significant difference between the specified groups, with # *p* < 0.05, ## *p* < 0.01, and ### *p* < 0.001 corresponding to different levels of significance.

To further investigate the mechanisms by which 1 μM RSL3 attenuates the regulatory effects of 0.5 mM NaB on lipid metabolism in 1 mM FFA-induced hepatocytes, the activity of cellular antioxidant enzymes, ROS content, and the protein expression of the GPX4-mediated ferroptosis pathway were assessed. As shown in Figure 5A, FFAs observably increased the accumulation of cellular ROS, whereas RSL3 further upregulated the accumulation of it (*p* < 0.05). Additionally, RSL3 decreased the ability of hepatocellular antioxidants, as evidenced by upregulating the content of MDA and GSH (Figure 5B,C). Moreover, the co-treatment of RSL3 and/or NaB significantly activated the GPX4-mediated ferroptosis signaling pathway compared to treatment with NaB alone, as evidenced by the downregulated protein expression of GPX4 and xCT, and the upregulated protein expression of FTH1 and COX-2 (Figure 5E,F).

### 3.6. The Ferroptosis Inhibitor Fer-1 Enhanced the Protective Effect of NaB in Mitigating FFAs-Induced Hepatic Steatosis

**Figure 6:**
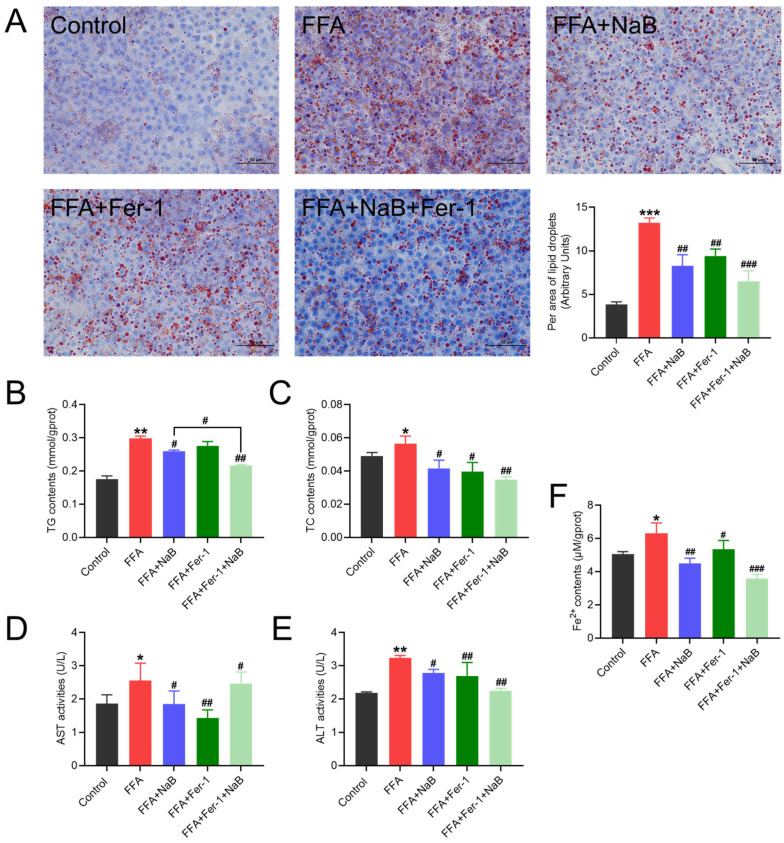
The ferroptosis inhibitor Fer-1 (5 μM) enhances the protection of NaB in mitigating FFAs-induced hepatic steatosis. (**A**) Effect of Fer-1 and/or NaB on lipid droplet accumulation in primary hepatocytes induced by FFAs (the lipid droplet area ratio was quantified using Image J). (**B**–**F**) Effect of Fer-1 and/or NaB on the contents of TG and TC, the activities of AST and ALT, and Fe^2+^ content in primary chicken hepatocytes induced by FFAs. An asterisk (*) denotes a statistically significant difference relative to the control group, with * *p* < 0.05, ** *p* < 0.01, and *** *p* < 0.001, indicating different levels of significance. A hash (#) signifies a significant difference between the specified groups, with # *p* < 0.05, ## *p* < 0.01, and ### *p* < 0.001 corresponding to different levels of significance.

To explore whether ferroptosis could serve as a potential therapeutic target for preventing and treating hepatic steatosis, Fer-1 (5 μM), an effective inhibitor of erastin-induced ferroptosis, was employed to intervene in the effects of 0.5 mM NaB on lipid metabolism in 1 mM FFAs-induced hepatocytes. The results indicated that Fer-1 effectively reduced the levels of TG and TC in FFAs-induced hepatocytes (*p* < 0.01), thereby diminishing the accumulation of lipid droplets in primary chicken hepatocytes induced by FFAs (*p* < 0.01) (Figure 6A–C). Moreover, Fer-1 further protected FFAs-mediated hepatocellular damage, as evidenced by the markedly decreased levels of ALT, AST, and Fe^2+^_,_ thereby ameliorating lipid-induced hepatocellular damage (*p* < 0.05) (Figure 6D–F).

### 3.7. Fer-1 Enhances the NaB-Mediated Antioxidative Response and GPX4 Expression in FFAs-Induced Hepatocytes to Mitigate GPX4-Mediated Ferroptosis

**Figure 7:**
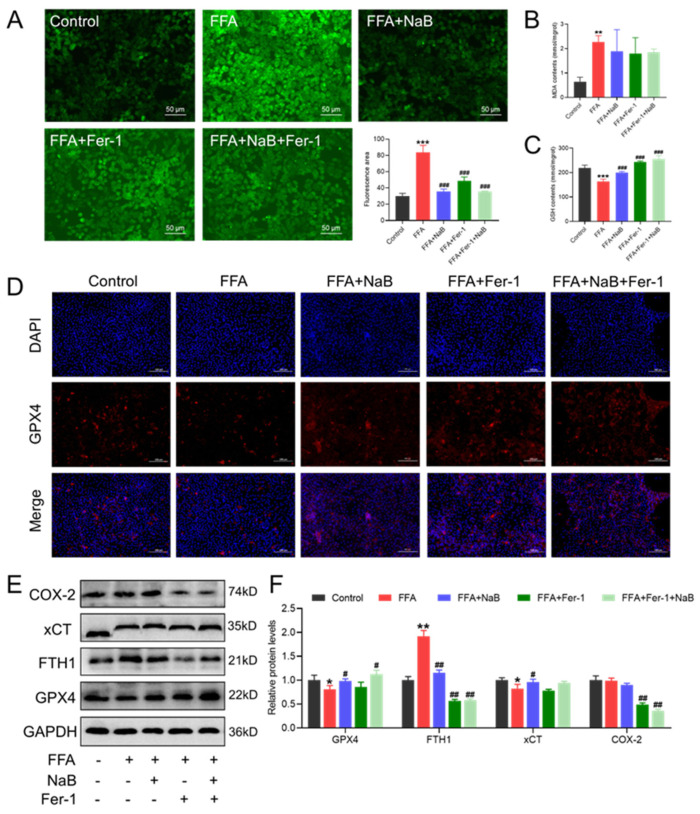
Fer-1 enhances NaB-mediated antioxidative response and GPX4 expression in FFAs-induced hepatocytes to mitigate GPX4-mediated ferroptosis. (**A**) Immunofluorescence images of the fluorescent probe DCFH-DA (ROS indicator, green) staining of representative cellular sections from various treatments (green fluorescence intensity was quantified using Image J). Scale bar: 50 μm. (**B**,**C**) Effect of Fer-1 and/or NaB on the contents of MDA and GSH in primary chicken hepatocytes induced by FFAs. (**D**) Immunofluorescence images of the fluorescent GPX4 (red) and DAPI (blue) staining of representative cellular sections from various treatments. Scale bar: 100 μm. (**E**,**F**) Effect of Fer-1 and/or NaB on the protein levels of COX-2, xCT, FTH1, and GPX4 in primary chicken hepatocytes induced by FFAs. An asterisk (*) denotes a statistically significant difference relative to the control group, with * *p* < 0.05, ** *p* < 0.01, and *** *p* < 0.001, indicating different levels of significance. A hash (#) signifies a significant difference between the specified groups, with # *p* < 0.05, ## *p* < 0.01, and ### *p* < 0.001 corresponding to different levels of significance.

To further elucidate the mechanisms underlying this promotion by 5 μM Fer-1 of 0.5 mM NaB-regulated lipid metabolism in 1 mM FFAs-induced hepatocytes, the activity of cellular antioxidant enzymes (GSH and MDA), ROS content, and the GPX4-mediated ferroptosis pathway were assessed. As shown in Figure 7A, FFAs observably increased the accumulation of cellular ROS, whereas Fer-1 downregulated the accumulation of it (*p* < 0.001). Additionally, Fer-1, akin to NaB, could enhance the ability of hepatocellular antioxidants, as evidenced by decreasing the content of MDA and increasing the content of GPX (*p* < 0.001), thereby attenuating ferroptosis. Additionally, the GPX4-mediated ferroptosis signaling pathway was significantly inhibited under combined treatment with Fer-1 and NaB in comparison with single-agent treatments, as evidenced by increasing the expression of GPX4 and decreasing the protein expression of xCT, FTH1, and COX-2 (Figure 7D–F).

## 4. Discussion

FLHS in laying hens is a nutritional metabolic disease commonly observed in high-yielding laying hens, causing significant economic losses to the global poultry farming industry [23]. Previous studies indicated that a reduction in butyrate production in laying hens from FLHS, and supplementation with NaB, could reduce hepatic steatosis, improving respiratory capacity and mitochondrial dysfunction [17,18]. Recently, ferroptosis has been an iron-dependent form of non-apoptotic cell death characterized by excessive lipid peroxidation and associated with a plethora of pathological conditions in the liver [26]. Emerging evidence indicates that ferroptosis may serve as a promising target for the prevention and treatment of various forms of liver disease [9,27]. In the present study, our results showed that NaB mitigates hepatic steatosis by lowering ROS and intracellular iron levels, which in turn modulates GPX4-mediated ferroptosis and subsequently reduces lipid accumulation. Importantly, the strategic manipulation of ferroptosis by employing inhibitors like Fer-1 or agonists such as RSL3 significantly sway the therapeutic effect of NaB on hepatic lipid accumulation, either augmenting or reducing its impact.

Butyrate, traditionally recognized as an energy substrate, is a short-chain fatty acid generated by the condensation of two acetyl-CoA molecules, forming acetoacetyl-CoA, which is then reduced to butyryl-CoA [28]. Recent investigations have indicated that NaB may mitigate hepatic steatosis, enhance respiratory capacity, and rectify mitochondrial dysfunction in obese mice [29]. Moreover, the administration of NaB in a protective coating prevented the accumulation of fat in the livers and abdominal fat tissues of broilers, while also significantly decreasing the buildup of adipocytic fat in chicken preadipocytes [19]. Our results also demonstrated that NaB effectively mitigates fatty acid-induced lipid accumulation and oxidative stress, as indicated by reduced ROS levels and upregulated antioxidant enzyme activities. Guo W. et al.’s studies revealed that butyrate can exert antioxidant and anti-apoptotic effects via the hydroxycarboxylic acid receptor 2 (GPR109A)/AMPK/nuclear factor erythroid2-related factor 2 (Nrf2) signaling pathway, where H3K9/14 acetylation promotes the transcription of Nrf2, thereby enhancing the antioxidant capacity in bovine mammary epithelial cells [30]. Additionally, Li L. et al. demonstrated that NaB ameliorates lipopolysaccharides-induced oxidative damage by upregulating the activities of antioxidative enzymes and reducing protein damage in MAC-T cells [31]. Additionally, the dietary supplementation of NaB can alleviate egg-laying stress in laying ducks by enhancing antioxidant ability [32]. The surplus of hepatic lipids can lead to alterations in cellular redox status, cumulating oxidative harm to biomacromolecules such as DNA, lipids, and proteins. Recent studies also showed that chronic exposure to lipotoxic species may abnormally stimulate an adaptive gene response, resulting in severe cellular changes, including mitochondrial damage or endoplasmic reticulum stress in liver cells [33]. Our findings indicate that lipid deposition in the liver induced by FFAs can lead to further mitochondrial impairment, as observed through transmission electron microscopy, while NaB can ameliorate these effects. The mitigation observed may be attributed to butyrate-enhanced antioxidative enzyme activity, improving hepatic lipid peroxidation and GSH metabolism. Previous studies have shown that NaB mitigates oxidative stress across diverse cellular matrices and enhances hepatic insulin signaling, culminating in the profound activation of the Nrf2 pathway, which orchestrates GSH metabolism and diminishes oxidative stress in C57BL/6J mice [34]. de-Cara A. et al.’s study found a linear and positive correlation between antioxidant enzymes and weight gain in broilers fed a NaB-supplemented diet [35]. Moreover, we observed that NaB can upregulate the expression of GPX4 and reduce iron levels in liver cells challenged with FFAs, suggesting that NaB could be involved in the regulatory mechanisms of ferroptosis, thereby influencing hepatic lipid metabolism.

Lipid peroxidation and ROS generation, consequent to iron-catalyzed Fenton reactions and enzymatic oxygenation, typify the ferroptosis-regulated cell death mechanism intimately associated with iron dysregulation and oxidative stress. Previous studies revealed that an abundance of redox-active iron catalyzes ROS via the Fenton reaction, instigating lipid peroxidation and ferroptosis induction [36]. Subsequent MDA formation from lipid peroxidation can be remediated by principal antioxidants such as GPX4 and superoxide dismutase, which mitigate MDA and ROS accumulation [37]. The diminution of GPX4 is critical in counteracting lipid peroxidation derivatives [38,39]. A previous study found that ferroptosis in the chicken brain mainly manifested as mitochondrial atrophy, increased Fe^2+^, decreased antioxidant enzyme activity, and the inhibition of the GPX4 expression level [40]. In the present study, our results found that FFAs-induced hepatic steatosis was associated with concurrent accumulations of iron and ROS, lipid peroxidation, and mitochondrial damage, alongside a reduction in GPX4 expression. GPX4, recognized as a crucial inhibitor of ferroptosis, enzymatically converts lipid hydroperoxides into non-toxic lipid alcohols, with GSH functioning as a cofactor [38]. The inhibition of GSH synthesis leads to GPX4 inactivation and consequent ferroptosis. Mao et al. revealed the roles of dihydroorotate dehydrogenase and mitochondrial GPX4 as defense mechanisms against mitochondrial lipid peroxides [41]. The fate of a cell undergoing ferroptosis is highly dependent on the coordinated regulation of ROS and lipid oxidation by subcellular organelles, including mitochondria, lysosomes, and peroxisomes. Zhao L. et al.’s study found that AFB1 induced cardiotoxicity and cardiomyocyte damage through ferroptosis-related signaling by increasing the level of ROS [42]. Our findings showed that fluctuations in ferroptosis-linked proteins such as FTH1, TFRC, and xCT are suggestive of ferroptosis activity. Moreover, NaB elevates GPX4 expression, curtails iron and ROS accumulation, and thus suppresses ferroptosis, potentially impeding lipid accumulation in hepatic cells.

Emerging evidence implicates ferroptosis as a pivotal contributor to the pathophysiology of an array of hepatic disorders including hemochromatosis, hepatitis C virus infection, and NAFLD [8,43]. As such, the modulation of ferroptosis represents a novel and promising therapeutic strategy for the amelioration of liver disease. Our investigations have revealed that NaB attenuates ferroptosis hallmarks in hepatocytes induced by FFAs, delineated by the mitigation of ROS proliferation, lipid peroxidative damage, and the downregulation of GPX4-mediated ferroptosis pathways, thereby alleviating hepatic lipid accumulation. However, the regulatory impact of NaB on ferroptosis was complex and not yet fully elucidated. A previous study from Wang GY showed that NaB promoted ferroptosis by inducing lipid ROS production via downregulating the expression of solute carrier family 7, membrane 11 (SLC7A11), and GPX4 through regulating the free fatty acid receptor 2 (FFAR2)-mammalian target of rapamycin (mTOR) signaling in colorectal cancer in HT29 cells [44]. Additionally, another research also suggested that NaB promoted ferroptosis in colorectal cancer cells through the CD44/SLC7A11 pathway [39]. Dietary coated sodium butyrate supplementation has a favorable effect in protecting against liver injury and alleviating lipid accumulation and inflammation by enhancing hepatic antioxidative function in aged laying hens [45]. Recently, studies revealed the diverse modulatory effects of butyrate on cellular metabolism, which exhibit a high degree of cell-type specificity [46]. The elucidated “butyrate paradox”, which describes butyrate’s divergent role in promoting cell proliferation in cancerous cells as a histone deacetylases inhibitor while simultaneously enhancing HAT1 activity in normal cell contexts, speaks to its dual functionality in humans [47,48]. To further delineate the regulatory mechanisms by which NaB modulates the ferroptosis signaling pathway, RSL-3, a potent class II ferroptosis inducer that mediates cell death by inactivating GPX4, was utilized in the present study. Our results showed that RSL-3 treatment effectively abrogates the protective effects of NaB on FFA-induced hepatic steatosis, and disrupts its influence on GPX4-mediated ferroptosis pathways. Moreover, the application of Fer-1, an agent notable for its capacity to scavenge radicals and ameliorate lipid peroxidation, enhanced the protective impact of NaB on FFA-induced hepatic steatosis. Interestingly, administration of Fer-1 alone yields outcomes akin to NaB treatment, which is in alignment with previous research findings. Intriguingly, treatment with Fer-1 alone can induce effects similar to those observed with NaB, which is in alignment with previous studies [49].

## 5. Conclusions

NaB effectively protects against FFAs-induced hepatocytic steatosis by modulating ROS/GPX4-mediated ferroptosis, and exhibits synergistic effects combined with Fer-1. These findings may provide novel perspectives for FLHS prevention and the co-administration of NaB with ferroptosis-inhibiting agents.

## Figures and Tables

**Table 1:** The primers for RT-qPCR assays.

Target Genes	Accession Number	Primers (5′-3′)
*GPX4*	NM_001346448.2	F: CAACGTGGCGTCCAAATGAG R: TCCACTTGATGGCATTCCCC
*TFRC*	NM_205256.2	F: GGAGACTCCTGATGCTATCGT R: TGGCATTTGCAACCTTCTCAG
*ACSL4*	XM_040700144.2	F: CTCAGCCATTTTAGCAGCCG R: CCAGCAGTGGACTCAAGGTA
*FTH1*	NM_205086.2	F: TTCCTGCGTCAACAGTGCTT R: CCGGTCAAAATAGTAGGACATGC
*NCOA4*	NM_001006495.2	F: CGTACCTTCGCCAGGCTATT R: CACACAGTGTTTTCTGCTGCT
*β-actin*	NM_205518.2	F: CAGCCAGCCATGGATGATGA R: CATACCAACCATCACACCCTGA

## Data Availability

The datasets analyzed during the current study are available from the corresponding author upon reasonable request.

## Abbreviations

ACSL4, Acyl-CoA synthetase long chain family member 4; AFLD, alcoholic fatty liver disease; ALT, alanine aminotransferase; AMPK, AMP-activated protein kinase; ANOVA, analysis of variance; AST, aspartate aminotransferase; BSA, bovine serum albumin; COX-2, cyclooxygenase-2; FBS, fetal bovine serum; Fer-1, ferrostatin-1; FLHS, fatty liver hemorrhagic syndrome; FFAs, free fatty acids; FFAR2, free fatty acid receptor 2; FTH1, ferritin heavy chain 1; GPR109A, hydroxycarboxylic acid receptor 2; GPX4, glutathione peroxidase 4; GSH, glutathione; GSH-pX, glutathione peroxidase; HELP, high-energy and low-protein; MDA, malondialdehyde; NaB, sodium butyrate; NAFLD, non-alcoholic fatty liver disease; NCOA4, nuclear receptor coactivator 4; Nrf2, nuclear factor erythroid2-related factor 2; OA, sodium oleate; PA, sodium palmitate; PBS, phosphate-buffered saline; ROS, reactive oxygen species; RSL3, RAS-selective lethal 3; TC, total cholesterol; TEM, transmission electron microscopy; TFRC, transferrin receptor; TG, triglyceride; xCT or SLC7A11, solute carrier family 7 member 11.
